# Predicting clinically significant medication errors: Development and validation of a risk stratification model using incident reporting data

**DOI:** 10.3389/fmed.2026.1887290

**Published:** 2026-09-01

**Authors:** Saad M. Wali, Nawaf S. Alqurashi, Malaz J. Gazzaz, Sharaf E. Sharaf, Maan H. Harbi, Fahad J. Alqahtani, Ohood K. Almuzaini, Eman O. Alsheikh, Saleh A. Almarshad, Sahar A. Abduljawad, Mohammed M. Aldurdunji

**Affiliations:** 1 Pharmacology and Toxicology Department, College of Pharmacy, Umm Al-Qura University, Makkah, Saudi Arabia; 2 Department of Pharmaceutical Care, Al-Noor Specialist Hospital, Makkah, Saudi Arabia; 3 Department of Pharmaceutical Practices, College of Pharmacy, Umm Al-Qura University, Makkah, Saudi Arabia; 4 Department of Pharmaceutical Sciences, College of Pharmacy, Umm Al-Qura University, Makkah, Saudi Arabia; 5 Department of Pharmacology and Toxicology, Faculty of medicine, Umm Al-Qura University, Al-Qunfudhah, Makkah, Saudi Arabia; 6 Department of Pharmacology and Toxicology, College of Pharmacy, Qassim University, Buraydah, Saudi Arabia; 7 Department of Health Administration and medical information, College of Al-leith Health Sciences, Umm Al-Qura University, Makkah, Saudi Arabia

**Keywords:** harm prevention, medication errors, medication safety, NCC MERP, patient safety, pharmacovigilance, predictive model, risk stratification

## Abstract

**Background:**

Medication error reporting systems capture large volumes of incidents, yet only a small proportion lead to clinically significant patient outcomes. Distinguishing these high-risk errors from the majority of low-impact events remains a major challenge for healthcare systems. This study aimed to develop a predictive model to identify medication errors at elevated risk of clinical significance in a tertiary care setting.

**Methods:**

A retrospective cross-sectional study was conducted at Al-Noor Specialist Hospital in Makkah, Saudi Arabia, analyzing medication error reports from January 2023 to August 2025. Clinically significant errors were defined as those reaching the patient with or without harm (NCC MERP Categories C–G). Univariate analyses identified candidate predictors, followed by multivariable logistic regression with category collapsing for model efficiency. Model performance was assessed using area under the receiver operating characteristic curve (AUC) and Brier score. Risk stratification divided predicted probabilities into low, moderate, and high-risk groups.

**Results:**

Among 6,397 medication errors, 227 (3.5%) were clinically significant. The final model demonstrated good discrimination (AUC = 0.825) and calibration (Brier score = 0.029). Independent predictors of clinically significant errors were high-risk error stages including administration, dispensing, and monitoring (OR = 19.1, 95% CI: 14.1–26.1), high-risk medication classes such as antibiotics and oncology agents (OR = 2.70, 95% CI: 1.62–4.78), intermediate or other medication classes (OR = 2.34, 95% CI: 1.39–4.18), selection or information errors (OR = 2.23, 95% CI: 1.40–3.46), and lack of staff experience (OR = 1.82, 95% CI: 1.33–2.49). Variables describing who detected or who committed the error were deliberately excluded from the model because they are not available at the moment an error occurs. Risk stratification classified 10.1% of errors as high-risk, capturing 62.6% of all clinically significant events.

**Conclusion:**

A parsimonious predictive model using readily available incident report data can effectively identify medication errors at elevated risk of clinical significance. The risk-stratification framework enables prioritization of safety resources toward high-risk incidents requiring immediate intervention.

## Introduction

Medication errors represent a persistent threat to patient safety across healthcare systems worldwide. The World Health Organization estimates that medication-related harm accounts for approximately $42 billion in annual global costs, prompting the launch of the Medication Without Harm initiative targeting a 50% reduction in severe avoidable medication-related harm (1). In the United States, medication errors are estimated to harm at least 1.5 million patients annually, contributing to prolonged hospitalizations, increased healthcare costs, and preventable mortality (2). While the majority of medication errors are intercepted before reaching patients or cause no measurable harm, a critical subset results in clinically significant outcomes requiring intervention, monitoring, or causing temporary or permanent patient injury (3, 4).

The ability to predict which medication errors will result in patient harm has important implications for healthcare quality improvement. Traditional approaches to medication safety have focused on error prevention across all incidents, regardless of potential severity. However, resource constraints necessitate prioritization, and evidence suggests that targeted interventions directed at high-risk errors may yield greater safety improvements than universal approaches (5, 6). Predictive models that identify characteristics associated with harmful outcomes could enable real-time risk stratification, allowing healthcare organizations to allocate safety resources more effectively and implement immediate interventions for incidents most likely to cause patient harm (7).

Research examining predictors of medication error severity has identified several consistent risk factors. Error stage plays a critical role, with errors occurring at administration and monitoring stages more likely to reach patients than those intercepted during prescribing or dispensing (8, 9). Certain medication classes, including high-alert medications such as anticoagulants, opioids, insulin, and chemotherapeutic agents, carry elevated risk of serious harm when errors occur (10, 11). System-level factors including staffing adequacy, workload, and healthcare professional experience have also been associated with error severity (12, 13). However, integrated predictive models combining patient, drug, and system factors to stratify medication error risk remain limited, particularly in Middle Eastern healthcare contexts.

In the Middle East and North Africa region, medication error research has expanded considerably, with systematic reviews documenting high prevalence rates and identifying contributing factors including workload, communication barriers, and system deficiencies (14, 15). The diverse healthcare workforce in MENA countries, often comprising professionals trained in different international systems, may introduce additional complexity in medication management (16). However, predictive modeling approaches for medication error harm have not been widely applied in the region, representing an important gap in the evidence base for safety improvement.

In Saudi Arabia, medication safety has become a national priority aligned with Vision 2030’s Health Sector Transformation Program (17). The Saudi Food and Drug Authority has established comprehensive pharmacovigilance infrastructure, and research has documented medication error patterns across Saudi healthcare facilities (18, 19). Studies have examined error characteristics and contributing factors, with prescribing errors predominating and pharmacists serving as primary detectors (20, 21). Nevertheless, predictive models specifically designed to identify errors at elevated risk of causing patient harm remain underdeveloped in Saudi healthcare settings, limiting the ability to implement targeted, risk-based safety interventions.

The primary objective of this study was to develop a predictive model for clinically significant medication errors, defined as those reaching the patient with or without harm according to National Coordinating Council for Medication Error Reporting and Prevention (NCC MERP) Categories C–G. Secondary objectives included identifying clinical, pharmacologic, and system-level factors most strongly associated with harmful outcomes, comparing prediction performance across modeling approaches, and constructing a risk-stratification framework to categorize medication errors into low, moderate, and high-risk groups for prioritization of safety interventions.

## Materials and methods

### Study design and setting

A retrospective cross-sectional study was conducted at Al-Noor Specialist Hospital in Makkah, Saudi Arabia. Al-Noor Specialist Hospital is a tertiary care center providing comprehensive medical, surgical, and specialized services to a diverse patient population. The hospital operates an electronic medication error reporting system that enables healthcare professionals to voluntarily report medication-related incidents.

### Study Period and Data Source

Data were extracted from medication error reports submitted to the hospital’s electronic reporting system between January 1, 2023, and August 31, 2025. The reporting system captures standardized information on each incident, including patient demographics, medication details, error characteristics, contributing causes, detection source, and outcome severity classified according to NCC MERP categories. Severity is assigned in two stages. The person who identifies the error submits the report and records a preliminary NCC MERP category at that point. The Medication Safety Officer subsequently investigates the incident and reviews that category; where the classification is revised, the original reporter is notified of the final assignment. The analyses reported here use the reviewed classification.

### Outcome definition

Due to the very small number of incidents resulting in direct injury (NCC MERP categories E–G), clinically significant harm was defined more broadly to include errors that reached the patient and required monitoring (Categories C–G). Category C includes errors that reached the patient but caused no harm, Category D includes errors requiring monitoring to confirm no harm, and Categories E–G include errors causing temporary harm, harm requiring hospitalization, or permanent harm, respectively (4).

### Predictor variables

Candidate predictor variables included error characteristics (stage, type, cause), medication class, error committer, and error detector. Two of these candidates were subsequently excluded from the predictive model on conceptual grounds. Error detector was excluded because the identity of the person who intercepts an error is established only after the error has occurred and been caught; it is a consequence of the event rather than a characteristic available at the moment the error is generated, and a model intended to score incoming reports must rely on information present at that point. Error committer was excluded because it overlapped substantially with error stage, nurses being predominantly involved in administration errors and physicians in prescribing. The final model therefore retains only variables describing the error itself and the circumstances of its occurrence. For model development, predictor variables were collapsed into clinically meaningful categories to improve model parsimony and stability based on observed associations and clinical rationale.

### Variable transformations

Medication classes were condensed into three risk groups: high-risk classes (antibiotics/antimicrobials and hematology/oncology/immunology drugs), low-risk classes (electrolytes/fluids/vitamins, endocrine/metabolic, and gastrointestinal/hepatic medications), and intermediate/other classes (remaining categories). This grouping was based on observed associations in univariate analysis, whereby antibiotics and oncology/immunology drugs showed the highest rates of clinically significant errors, while electrolytes, endocrine, and gastrointestinal drugs showed the lowest. Error stage was transformed into a binary indicator reflecting likelihood of patient exposure: high-risk stages (administration, dispensing, monitoring) versus intercepted stages (prescribing, transcribing, preparation). Error types were grouped as higher-risk mechanisms (selection and information/documentation errors) versus lower-risk mechanisms (dosing, duplication, and other errors). For error causes, only lack of staff experience was retained as a binary predictor given its strong association with clinically significant outcomes. Error detector was grouped into pharmacy team (pharmacists or assistant pharmacists), clinical staff (physicians or nurses), and patient/other.

### Statistical analysis

All statistical analyses were performed using RStudio (version 2024.9.1.394, Boston, MA, USA) with R version 4.4.2. Categorical variables were summarized using frequencies and percentages and compared using Pearson’s chi-squared or Fisher’s exact test, as appropriate. To identify factors associated with clinically significant medication errors, univariate analyses were first conducted, followed by multivariable logistic regression. Category collapsing was performed to avoid sparse data bias and model overfitting because the relatively small number of clinically significant events did not support reliable estimation across all original categorical levels. Categories were merged according to clinical similarity and their observed associations with the outcome in the inferential analyses. Model discrimination was evaluated using the area under the receiver operating characteristic (ROC) curve (AUC). Model calibration, reflecting the agreement between predicted probabilities and observed outcomes, was assessed using the Brier score, with lower values indicating better calibration. Variance inflation factors were calculated to assess multicollinearity. Risk stratification was conducted by dividing predicted probabilities into three strata (low, moderate, and high risk) based on percentile thresholds. A *p*-value of < 0.05 was considered statistically significant.

### Ethical considerations

Ethical approval for this study was obtained from the Institutional Review Board (IRB) of the Local Committee for Research Ethics in Makkah (IRB No.: H-02-K-076-0226-1551). The research was conducted in accordance with the ethical standards of the Declaration of Helsinki (22) and complied with relevant data protection regulations to maintain patient confidentiality (23). Given the retrospective design and the use of de-identified data, the requirement for informed consent was waived by the IRB.

## Results

### Demographic and error-related characteristics of patients and their association with clinically significant errors

Among the 6,397 reported medication error incidents, most involved adult patients (92.3%), and 54.6% involved male patients. Most errors were committed by physicians (95.5%) and detected by pharmacists (86.4%). Antibiotics/antimicrobials were the most frequently involved medication class (33.2%), followed by cardiovascular drugs (22.7%). Prescribing was the most common error stage (65.6%), and dosing errors were the predominant error type (66.7%). The most frequently cited causes were environmental factors (41.1%) and lack of staff experience (19.7%, Table 1).

**TABLE 1:** Demographic and error-related characteristics of patients and their association with clinically-significant errors.

		Clinically-significant error		
Characteristic	Overall *N* = 6,397	No clinically significant error *N* = 6170	Clinically significant error *N* = 227	*P*-value
Age				0.380
Pediatric (≤18 years)	492 (7.7%)	478 (7.7%)	14 (6.2%)	
Adult (>18 years)	5,905 (92.3%)	5,692 (92.3%)	213 (93.8%)	
Gender				0.499
Male	3,494 (54.6%)	3,375 (54.7%)	119 (52.4%)	
Female	2,903 (45.4%)	2,795 (45.3%)	108 (47.6%)	
Error is committed by				
Pharmacist	91 (1.4%)	77 (1.2%)	14 (6.2%)	<0.001
Assistant pharmacist	111 (1.7%)	109 (1.8%)	2 (0.9%)	0.440
Nurse	72 (1.1%)	36 (0.6%)	36 (15.9%)	<0.001
Physician	6,106 (95.5%)	5,934 (96.2%)	172 (75.8%)	<0.001
Patient	2 (0.0%)	1 (0.0%)	1 (0.4%)	0.070
Others	17 (0.3%)	13 (0.2%)	4 (1.8%)	0.003
Error is detected by				
Physician	57 (0.9%)	37 (0.6%)	20 (8.8%)	<0.001
Pharmacist	5,526 (86.4%)	5,364 (86.9%)	162 (71.4%)	<0.001
Assistant pharmacist	694 (10.8%)	691 (11.2%)	3 (1.3%)	<0.001
Nurse	93 (1.5%)	58 (0.9%)	35 (15.4%)	<0.001
Patient/caregiver	9 (0.1%)	4 (0.1%)	5 (2.2%)	<0.001
Others	18 (0.3%)	16 (0.3%)	2 (0.9%)	0.132
Broad category				
Antibiotics / antimicrobials	2,121 (33.2%)	2,002 (32.4%)	119 (52.4%)	<0.001
Cardiovascular drugs	1,451 (22.7%)	1,399 (22.7%)	52 (22.9%)	0.934
CNS / psychiatric / neurologic	152 (2.4%)	143 (2.3%)	9 (4.0%)	0.110
Electrolytes, fluids, vitamins, supplements	825 (12.9%)	819 (13.3%)	6 (2.6%)	<0.001
Endocrine and metabolic	454 (7.1%)	447 (7.2%)	7 (3.1%)	0.016
Gastrointestinal and hepatic	318 (5.0%)	314 (5.1%)	4 (1.8%)	0.024
Hematology / oncology / immunology	236 (3.7%)	221 (3.6%)	15 (6.6%)	0.018
Pain, anti-inflammatory, and anesthesia	518 (8.1%)	507 (8.2%)	11 (4.8%)	0.067
Respiratory	184 (2.9%)	182 (2.9%)	2 (0.9%)	0.067
Other / miscellaneous	138 (2.2%)	136 (2.2%)	2 (0.9%)	0.243
Error stage				
Administration	79 (1.2%)	42 (0.7%)	37 (16.3%)	<0.001
Prescribing	4,195 (65.6%)	4,109 (66.6%)	86 (37.9%)	<0.001
Transcribing	1,689 (26.4%)	1,676 (27.2%)	13 (5.7%)	<0.001
Preparing	99 (1.5%)	98 (1.6%)	1 (0.4%)	0.267
Monitoring	279 (4.4%)	209 (3.4%)	70 (30.8%)	<0.001
Dispensing	56 (0.9%)	36 (0.6%)	20 (8.8%)	<0.001
Error type				
Dosing errors	4,267 (66.7%)	4,140 (67.1%)	127 (55.9%)	<0.001
Selection errors	126 (2.0%)	118 (1.9%)	8 (3.5%)	0.089
Information errors	338 (5.3%)	317 (5.1%)	21 (9.3%)	0.007
Duplication errors	913 (14.3%)	882 (14.3%)	31 (13.7%)	0.787
Other errors	753 (11.8%)	713 (11.6%)	40 (17.6%)	0.005
Error cause				
System causes	1,197 (18.7%)	1,154 (18.7%)	43 (18.9%)	0.928
Environmental causes	2,626 (41.1%)	2,605 (42.2%)	21 (9.3%)	<0.001
Error cause	1,261 (19.7%)	1,155 (18.7%)	106 (46.7%)	<0.001
*n* (%)				

Pearson’s chi-squared test; Fisher’s exact test.

### Frequencies and proportions of clinically significant errors

**FIGURE 1:**
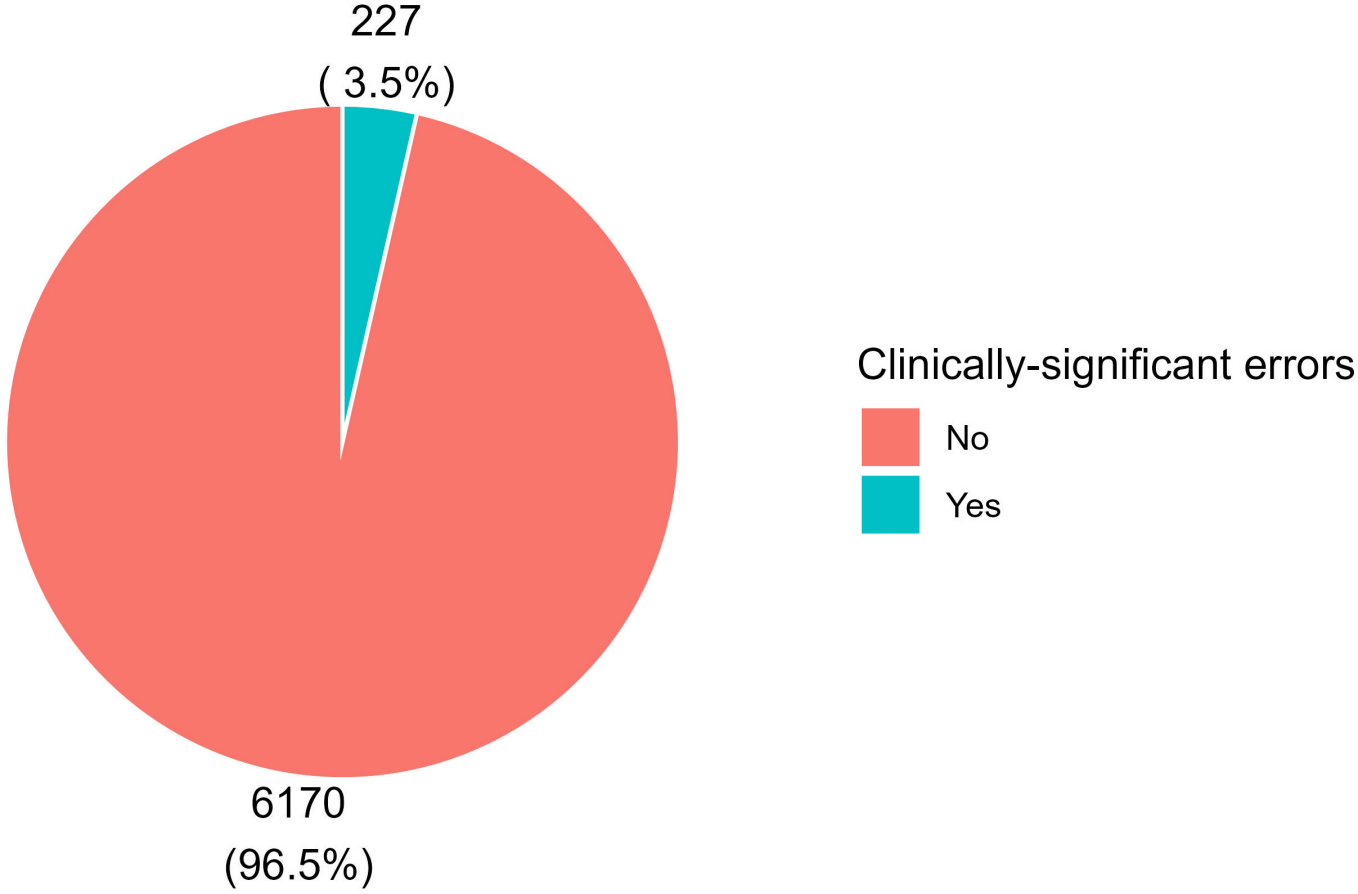
Frequencies and proportions of clinically-significant errors.

Out of 6,397 medication error incidents, 201 (3.1%) reached the patient without causing harm (Category C), while 23 (0.4%) required monitoring (Category D). Errors resulting in temporary harm (Categories E and F) and permanent harm (Category G) were rare, each accounting for one incident. Overall, clinically significant errors (Category C to G) accounted for 3.5% of the errors reported in the current study (Figure 1).

### Factors associated with clinically significant errors

Clinically significant errors were significantly less likely to be committed by physicians (75.8% vs. 96.2%, *p* < 0.001) and more likely to be committed by nurses (15.9% vs. 0.6%, *p* < 0.001) and pharmacists (6.2% vs. 1.2%, *p* < 0.001). Errors detected by physicians (8.8% vs. 0.6%, *p* < 0.001), nurses (15.4% vs. 0.9%, *p* < 0.001), and patients or caregivers (2.2% vs. 0.1%, *p* < 0.001) were more likely to be clinically significant. Conversely, errors detected by pharmacists (71.4% vs. 86.9%, *p* < 0.001) and assistant pharmacists (1.3% vs. 11.2%, *p* < 0.001) were less likely to be clinically significant.

Antibiotics/antimicrobials (52.4% vs. 32.4%, *p* < 0.001) and hematology/oncology/immunology drugs (6.6% vs. 3.6%, *p* = 0.018) were more frequently associated with clinically-significant errors, whereas low-risk medication classes such as electrolytes/fluids/vitamins (2.6% vs. 13.3%, *p* < 0.001), endocrine/metabolic agents (3.1% vs. 7.2%, *p* = 0.016), and gastrointestinal/hepatic drugs (1.8% vs. 5.1%, *p* = 0.024) were less frequently involved.

Clinically significant errors occurred more often during administration (16.3% vs. 0.7%, *p* < 0.001), monitoring (30.8% vs. 3.4%, *p* < 0.001), and dispensing (8.8% vs. 0.6%, *p* < 0.001) stages. In contrast, prescribing (37.9% vs. 66.6%, *p* < 0.001) and transcribing errors (5.7% vs. 27.2%, *p* < 0.001) were less likely to be clinically significant.

Among error types, dosing errors were less common in clinically significant events (55.9% vs. 67.1%, *p* < 0.001), while information errors (9.3% vs. 5.1%, *p* = 0.007) and other error types (17.6% vs. 11.6%, *p* = 0.005) were more common. Errors attributed to a lack of staff experience were significantly associated with clinically significant outcomes (46.7% vs. 18.7%, *p* < 0.001), whereas environmental causes were less frequently observed (9.3% vs. 42.2%, *p* < 0.001, Table 1).

### Predictive modeling

**FIGURE 2:**
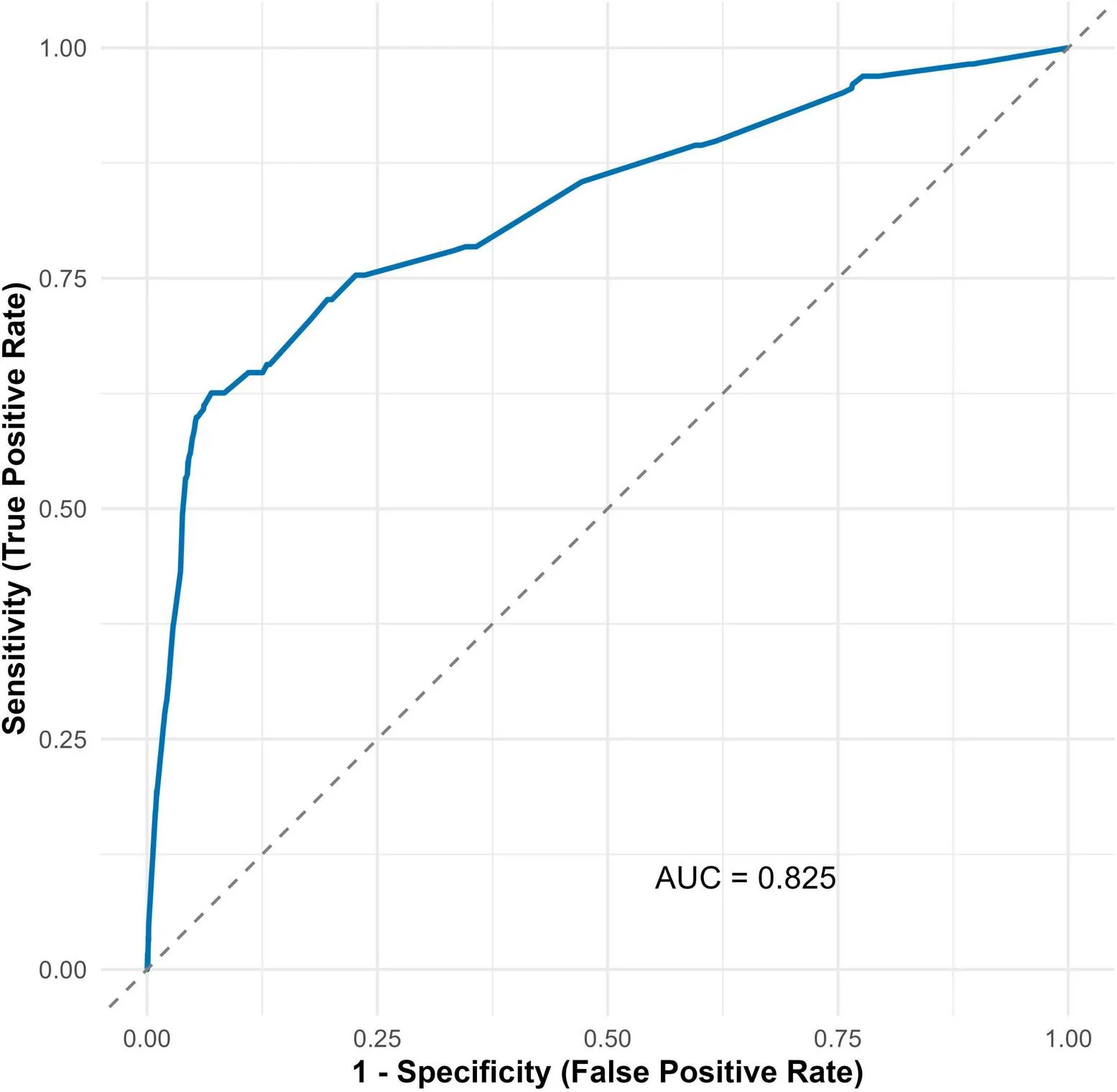
ROC curve for clinically-significant error prediction.

Although large differences were observed in who committed the errors, these categories might have strongly overlapped with error stage (e.g., nurses commonly involved in administration errors; physicians in prescribing). The final multivariable logistic regression model showed good overall discrimination, with an area under the ROC curve (AUC) of 0.825 (Figure 2). The model also demonstrated good calibration, with a Brier score of 0.029, indicating close agreement between the predicted probabilities and the observed occurrence of clinically significant medication errors. There was no risk of multicollinearity (variance inflation factor values were less than 5 for all predictor variables).

### Predictors of clinically significant medication errors

In the multivariable logistic regression model, intermediate/other medication classes (OR = 2.34, 95% CI 1.39–4.18, *p* = 0.002) and high-risk medication classes (OR = 2.70, 95% CI 1.62–4.78, *p* < 0.001) were significantly associated with increased odds of clinically significant medication errors compared to low-risk medication classes. Errors occurring in high-risk stages (administration, monitoring, dispensing) demonstrated substantially greater odds of clinical significance (OR = 19.1, 95% CI 14.1–26.1, *p* < 0.001) relative to intercepted stages. Selection or information errors were also significantly associated with clinically significant harm (OR = 2.23, 95% CI 1.40–3.46, *p* < 0.001), as were errors caused by lack of staff experience (OR = 1.82, 95% CI 1.33–2.49, *p* < 0.001, Table 2).

**TABLE 2:** Predictors of clinically-significant medication errors.

Characteristic	OR	95% CI	*P*-value
Age group			
Pediatric (≤18 years)	Reference	Reference	
Adult (>18 years)	0.95	0.55, 1.78	0.868
Gender			
Male	Reference	Reference	
Female	1.13	0.85, 1.52	0.397
Medication risk group			
Low-risk medication classes (electrolytes, endocrine, GI)	Reference	Reference	
Intermediate/other medication classes	2.34	1.39, 4.18	0.002
High-risk medication classes (antibiotics, oncology/immunology)	2.70	1.62, 4.78	<0.001
Error stage risk			
Intercepted	Reference	Reference	
High	19.1	14.1, 26.1	<0.001
Error type risk			
Dosing/duplication/other error types	Reference	Reference	
Selection/information errors	2.23	1.40, 3.46	<0.001
Error cause			
System/environmental causes	Reference	Reference	
Lack of staff experience	1.82	1.33, 2.49	<0.001

CI, confidence interval, OR, odds ratio.

### Characteristics of medication errors by predicted risk group

**FIGURE 3:**
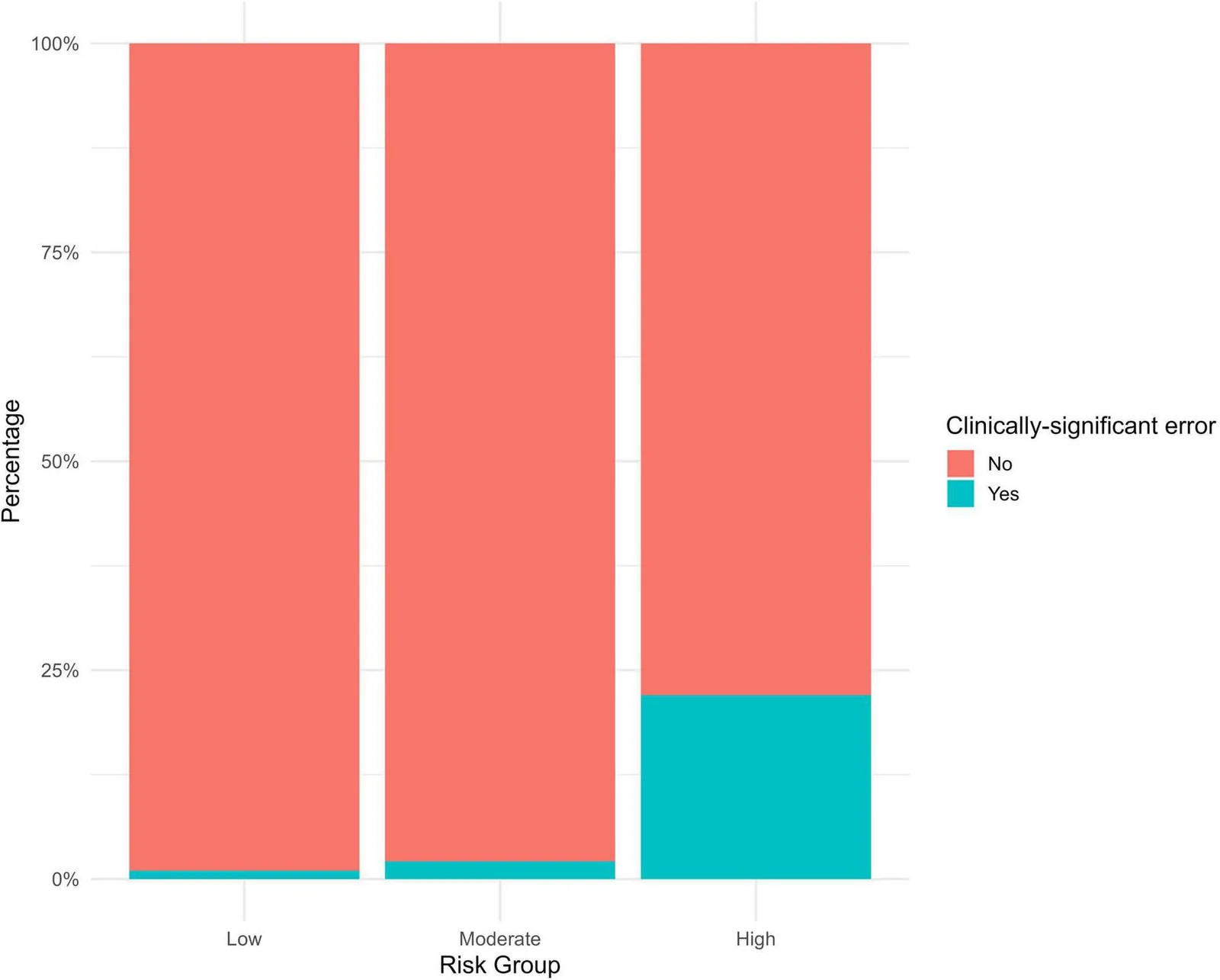
Proportions of clinically-significant errors by risk group.

Predicted probabilities generated from the final multivariable logistic regression model were used to classify the 6,397 medication error reports into three predefined risk strata based on percentile cut-offs: low risk (<60th percentile), moderate risk (60th–90th percentile), and high risk (>90th percentile). Across the three predicted risk groups, low-risk incidents comprised 3,291 (51.4%) reports, moderate-risk 2,460 (38.5%), and high-risk 646 (10.1%). Low-risk medication classes predominated in the low-risk group (47.3%) but were rare in the moderate-risk group (0.2%) and sparse in the high-risk group (5.4%). Conversely, high-risk medication classes were minimal in the low-risk group (0.0%), dominant in the moderate-risk group (79.5%), and comprised 62.2% of the high-risk group. Detection by the pharmacy team was nearly universal in the low- and moderate-risk groups (98.5% and 98.6%, respectively), but declined to 85.6% in the high-risk group, where clinical staff accounted for 12.5% and patients or others for 1.9% of detections. System or environmental causes were the predominant error cause in the low-risk group (95.0%), while lack of staff experience became more prevalent in the moderate-risk (33.8%) and high-risk (40.9%) groups. Clinically significant medication errors increased substantially across risk strata: 1.0% in the low-risk group, 2.1% in the moderate-risk group, and 22.0% in the high-risk group (Table 3). The proportion of clinically significant errors increased progressively across the risk strata, from 1.0% in the low-risk group to 2.1% in the moderate-risk group and 22.0% in the high-risk group (Figure 3).

**TABLE 3:** Characteristics of medication errors by predicted risk group.

Characteristic	Predicted risk group		
	Low *N* = 3291	Moderate *N* = 2460	High *N* = 646
Medication classes			
Low-risk medication classes (electrolytes, endocrine, GI)	1,558 (47.3%)	4 (0.2%)	35 (5.4%)
Intermediate/other medication classes	1,733 (52.7%)	501 (20.4%)	209 (32.4%)
High-risk medication classes (antibiotics, oncology/immunology)	0 (0.0%)	1,955 (79.5%)	402 (62.2%)
Error is detected by			
Pharmacy team (pharmacist/assistant pharmacist)	3,241 (98.5%)	2,426 (98.6%)	553 (85.6%)
Clinical staff (physicians/nurses)	37 (1.1%)	32 (1.3%)	81 (12.5%)
Patient/other	13 (0.4%)	2 (0.1%)	12 (1.9%)
Error cause			
System/environmental causes	3,125 (95.0%)	1,629 (66.2%)	382 (59.1%)
Lack of staff experience	166 (5.0%)	831 (33.8%)	264 (40.9%)
Clinically-significant errors			
No	3,258 (99.0%)	2,408 (97.9%)	504 (78.0%)
Yes	33 (1.0%)	52 (2.1%)	142 (22.0%)
*n* (%)			

## Discussion

This study developed a predictive model for clinically significant medication errors using 6,397 incident reports from a Saudi tertiary care hospital. The model demonstrated good discrimination (AUC = 0.825) and calibration (Brier score = 0.029), indicating reliable identification of errors at elevated risk of reaching patients. Four groups of predictors were independently associated with clinically significant outcomes: occurrence at high-risk stages (administration, dispensing, monitoring), involvement of high-risk or intermediate medication classes, selection or information error types, and errors attributed to lack of staff experience. All are properties of the error itself or of the circumstances in which it arose, and all are recorded at the time a report is submitted. The risk-stratification framework effectively concentrated harm within a targeted subset, with the high-risk group (10.1% of all errors) capturing 62.6% of all clinically significant events; within that group 22.0% of incidents were clinically significant, compared with 1.0% in the low-risk group.

### Interpretation of key predictors

Errors detected by clinical staff (physicians and nurses) rather than by pharmacy teams were substantially more likely to be clinically significant in bivariate analysis (Table 1), and pharmacy involvement in detection fell from 98.5% in the low-risk group to 85.6% in the high-risk group (Table 3). This association is descriptive rather than predictive. Detection is established only once an error has occurred and been intercepted, so it cannot serve to flag an error prospectively; an error that escapes pharmacy review cannot, by definition, be identified by pharmacy. Detection source was therefore excluded from the predictive model. What the observation does support is the role of pharmacy verification as an interception point in the medication-use process, since the errors that progressed toward the patient without pharmacy involvement were those most often associated with clinical significance. The implication is an argument for extending pharmacy coverage across the medication-use process, particularly at administration and monitoring, rather than for treating detection source as a warning signal (24, 25).

The strong association between high-risk error stages and clinical significance (OR = 19.1) aligns with the conceptual framework of medication error interception. Errors occurring during prescribing and transcribing represent opportunities for downstream detection, whereas errors at administration, dispensing, and monitoring stages have already progressed through multiple verification steps (26). The administration stage, in particular, represents the final opportunity for error detection before patient exposure. This finding supports targeted interventions at high-risk stages, including barcode medication administration systems, independent double-checks for high-alert medications, and enhanced monitoring protocols (27, 28).

The elevated risk associated with antibiotics and oncology/immunology drugs (OR = 2.70) is consistent with their designation as high-alert medication categories. Antibiotics involve complex dosing calculations, renal adjustments, and therapeutic monitoring requirements that create opportunities for error (29). Oncology agents carry narrow therapeutic indices and severe toxicity profiles that amplify the consequences of errors (30). The identification of these classes as independent predictors of harm supports prioritization of safety interventions for these therapeutic categories, including specialized training, clinical decision support, and enhanced verification protocols.

The association between lack of staff experience and clinically significant errors (OR = 1.82) highlights the importance of training, supervision, and competency assessment in medication safety. Inexperienced staff may lack familiarity with medication protocols, fail to recognize potential errors, or be more susceptible to workflow disruptions (31). An important qualification applies here: lack of staff experience is a contributing cause recorded by the reporter at the time of the incident, not a formal assessment of competency, and the two are related but not equivalent. An experienced practitioner may still err under conditions of interruption, fatigue, or high workload, while an inexperienced one may perform reliably within a well-designed system. Staffing levels, workflow design, interruptions, and the availability of supervision plausibly contribute alongside individual inexperience, but the reporting system records a single contributing cause per incident and cannot disentangle these; the association should therefore be read as a signal about the circumstances under which errors arise rather than as a judgment about individual practitioners. This finding supports targeted onboarding programs, mentorship arrangements, and competency-based training for medication administration, alongside attention to the working conditions in which medication is prepared and given.

### Risk Stratification for safety prioritization

The risk-stratification framework demonstrated the model’s practical utility for safety prioritization. The high-risk group, comprising only 10.1% of all reported incidents, captured 62.6% of all clinically significant errors (142 of 227). Within this group, 22.0% of incidents were clinically significant–approximately 22 times higher than the 1.0% rate observed in the low-risk group. This concentration of harm within a targeted subset enables efficient resource allocation, allowing safety teams to focus immediate attention on incidents most likely to cause patient harm while applying standard review processes to lower-risk events (32).

The characteristics of each risk group provide actionable guidance for intervention design. Low-risk incidents were predominantly detected by pharmacy teams, involved lower-risk medications, and rarely resulted from staff inexperience–suggesting that existing safety systems adequately manage these events. High-risk incidents, conversely, were characterized by clinical staff detection, high-risk medications, and staff inexperience, representing clear targets for enhanced intervention including real-time alerts, mandatory verification protocols, and root cause analysis (33).

### Comparison with prior literature

These findings extend previous research on medication error severity predictors. Studies in Western healthcare settings have similarly identified administration stage errors, high-alert medications, and inexperienced staff as risk factors for harm (8, 10, 13). Concordance rather than divergence was expected. The medication-use process follows a broadly similar sequence of prescribing, verification, dispensing, and administration across health systems, and the mechanisms by which an error reaches a patient are correspondingly comparable; there is no *a priori* reason why the direction of these associations should differ. What varies between settings is their magnitude and the organizational context in which they arise, including staffing models, the extent of pharmacy involvement in verification, and the composition of a workforce trained in different international systems. The current study adds to this evidence base by demonstrating the predictors in a Middle Eastern healthcare context and by developing an integrated risk-stratification model suitable for operational implementation.

The model’s AUC of 0.825 indicates good discriminative ability, meaning the model correctly ranks a randomly selected clinically significant error higher than a randomly selected non-significant error approximately 83% of the time. A recent meta-analysis of machine learning models for adverse drug event prediction reported a pooled AUC of 0.72 (95% CI: 0.68–0.75), with individual studies showing considerable variation depending on outcome definition and predictor availability (34). The parsimonious approach using collapsed categories enhances the model’s practical applicability, as the required predictors are routinely captured in incident reporting systems.

### Clinical and policy implications

The predictive model and risk-stratification framework have several practical applications for medication safety improvement. First, the model could be integrated into electronic incident reporting systems to generate real-time risk scores, flagging high-risk incidents for immediate review and intervention. Second, the identified predictors can guide targeted safety initiatives, including enhanced verification protocols for high-risk medications, competency-based training programs for inexperienced staff, and workflow redesign to strengthen pharmacy interception of errors. Third, the risk-stratification framework enables efficient allocation of limited safety resources, directing intensive investigation toward incidents most likely to cause harm while maintaining standard processes for lower-risk events. In operational terms, the model is applied to an incident report at the point of submission. Each of its predictors – error stage, medication class, error type and reported contributing cause – is a field already completed by the reporter, so a risk score can be generated automatically without additional data entry and without waiting for review. The score does not replace severity classification; it orders the review queue. This is where its relationship to the preliminary category matters. The category recorded at submission is assigned by the person who identified the error, before investigation, and is revised by the Medication Safety Officer where review shows it to be inaccurate. The model score is computed from objective characteristics of the error – stage, medication class, error type and reported cause – and is therefore independent of that initial judgment. A report whose preliminary category understates its risk, but whose characteristics place it in the high-risk stratum, would be brought forward for earlier investigation rather than waiting in the queue behind reports that were categorized more conservatively at submission. Where reporting volume exceeds the capacity of the safety team to investigate every report promptly, or where formal severity coding is assigned later in the review process, the score identifies which reports should be examined first. On the present data, prioritizing the 10.1% of reports classified as high risk would bring 62.6% of all clinically significant errors forward for immediate review. Its second use is aggregate rather than individual: because the model quantifies how much each factor contributes and how they combine, it identifies the specific combinations – for example a high-risk medication class reaching the administration stage – that concentrate harm within this hospital, which is the level at which preventive barriers are designed. It should be acknowledged that the individual predictors identified here are consistent with established knowledge in medication safety, and the study does not claim to have discovered new risk factors. Its contribution is of a different kind: these factors are combined into a single calibrated score with quantified discrimination, which converts a list of known risks into a ranking of individual reports. A safety team already knows that administration-stage errors involving high-alert medications are dangerous; what it does not know, faced with several hundred reports in a month, is which of them to open first. That is the gap the model addresses. Prioritization is not the same as dismissal. Consistent with a systems view of error causation, an incident that caused no harm on one occasion may recur under different circumstances and with a different medication, and low-risk reports retain their value for aggregate analysis, trend detection and the identification of latent conditions. The stratification proposed here is intended to sequence investigative effort when resources are finite, not to define which reports deserve attention.

At the policy level, these findings support investment in pharmacy infrastructure as a cornerstone of medication safety systems. The observation that errors progressing beyond pharmacy verification were disproportionately those of clinical significance suggests that adequate pharmacist staffing and workflow designs that maximize pharmacy involvement in medication verification should be prioritized (35); this inference rests on the descriptive comparison rather than on the predictive model, from which detection source was excluded. Additionally, the association between staff inexperience and harm supports policies requiring structured training and competency assessment before independent medication administration responsibilities.

### Strengths and limitations

This study has several strengths. The large sample size (*n* = 6,397) provides adequate statistical power for model development. The use of category collapsing based on clinical rationale and observed associations enhances model parsimony and interpretability. The assessment of both discrimination (AUC) and calibration (Brier score) provides comprehensive evaluation of model performance. The risk-stratification framework translates statistical predictions into operationally meaningful categories.

Several limitations should be acknowledged. First, the study relies on voluntary error reporting, which likely underestimates true error rates and may introduce reporting bias toward more severe events. Second, the single-center design limits generalizability to other healthcare settings. Third, the low overall rate of clinically significant errors (3.5%) necessitated a broader outcome definition including Category C (reached patient, no harm), which may limit the model’s specificity for predicting actual patient harm. While Category C errors do not involve harm, they represent failures of interception and therefore remain highly relevant for preventive safety strategies. Fourth, the model was developed and evaluated within a single dataset and has not been validated prospectively or in an external setting, so its performance elsewhere is unknown. Because the coefficients were estimated on the case mix, formulary and staffing profile of one hospital, another institution should not adopt the score as published; the appropriate use is to re-estimate it locally on that institution’s own reports, for which the required fields are those routinely captured by standard reporting systems. Prospective evaluation would involve applying the score to incoming reports over a defined period, comparing the resulting priority ranking with the severity subsequently assigned on review, and assessing whether high-risk reports were opened sooner and whether that changed what was found. Fifth, the retrospective design precludes prospective validation; future studies should assess model performance in independent datasets. Fifth, the model does not incorporate real-time clinical parameters (e.g., patient acuity, concurrent medications) that may influence harm probability but are not routinely captured in incident reports.

### Future directions

Future research should validate this predictive model in multi-center studies across diverse Saudi healthcare settings to assess generalizability. Prospective implementation studies could evaluate the impact of real-time risk stratification on safety outcomes and resource allocation efficiency. Machine learning approaches, including random forests and gradient boosting, could be compared with logistic regression to assess whether more complex models improve prediction performance. Additionally, qualitative research exploring healthcare professional perspectives on risk factors and intervention acceptability may inform implementation strategies. A larger multi-center dataset would also permit finer categorization of the predictor variables. In the present analysis the number of clinically significant events constrained how many categories could be estimated reliably, requiring several clinically distinct groups to be collapsed; a greater number of events would allow individual medication classes, error stages and error types to be modeled separately and their relative contributions distinguished.

## Conclusion

This study developed a predictive model demonstrating good discrimination for identifying medication errors at elevated risk of clinical significance. Key predictors included occurrence at administration, dispensing, or monitoring stages, involvement of antibiotics or oncology agents, selection or information error types, and errors attributed to lack of staff experience; all are recorded at the time a report is submitted, so the score can be generated without additional data collection. The risk-stratification framework effectively concentrated harm within a targeted high-risk subset comprising 10.1% of incidents, enabling prioritization of safety resources. Integration of this model into incident reporting systems could enhance medication safety by directing immediate attention toward errors most likely to cause patient harm, while the identified predictors provide actionable targets for system-level safety interventions.

## Data Availability

The raw data supporting the conclusions of this article will be made available by the authors, without undue reservation.
